# Effect of visual feedback during ultrasound-guided hydrodissection for myofascial pain syndrome: An exploratory, prospective, observational clinical trial on the expectations for treatment

**DOI:** 10.3389/fpsyt.2022.794425

**Published:** 2022-08-22

**Authors:** Hideaki Hasuo, Hideya Oomori, Kohei Yoshida, Mikihiko Fukunaga

**Affiliations:** Department of Psychosomatic Medicine, Kansai Medical University, Hirakata, Japan

**Keywords:** expectation, visual feedback, ultrasound-guide, interfascial injection, myofascial pain syndrome

## Abstract

Expectations for treatment have a favorable effect on the subsequent course of pain and behavior in patients. It is not known whether receiving hydrodissection while patients view their ultrasound image with doctors (visual feedback) is associated with positive treatment expectations. This was an exploratory, prospective, observational clinical trial. We explored the possibility that visual feedback immediately after ultrasound-guided hydrodissection increases the expectations for treatment, which could be one of the related factors for pain reduction. Treatment expectations were set as mediators of pain using path analysis. The primary endpoint was the numerical rating scale to assess expectations for treatment immediately after hydrodissection, between with and without the visual feedback. During 2019 and 2020, 136 outpatients received ultrasound-guided hydrodissection for myofascial pain syndrome. Of these, 65 (47.8%) patients received visual feedback during ultrasound-guided hydrodissection. Compared with the non-visual feedback group, the visual feedback group had higher expectations for treatment immediately after hydrodissection, and their expectations were maintained at day 14 of treatment (*p* < 0.001). A numerical rating scale (NRS) to assess expectations for treatment was similar before hydrodissection and immediately after hydrodissection was 8.4 (standard deviations, 1.6) in the visual feedback and 5.9 (standard deviations, 2.6) in the visual feedback. The proportion of increased expectations immediately after hydrodissection was 90.8% (95% CI: 83.7–97.9) in visual feedback group and 38.0% (95% CI: 26.7–49.3) in non-visual feedback group (*p* < 0.001). In the visual feedback group, 67.7% of patients showed improvement in pain numerical rating scale score by 50% or more at day 14, whereas such improvement was observed in only 36.6% of the non-visual feedback group (*p* < 0.001). Based on path analysis, the visual feedback had the greatest influence on pain numerical rating scale reduction at 14 days, indirectly due to increased expectations for treatment as a mediator (β = 0.434). However, the adjusted *R*^2^ values, which is the overall fit of the model, was low at 0.298. Visual feedback during ultrasound-guided hydrodissection increases the expectations for treatment immediately after hydrodissection, which could be one of the related factors for pain reduction in patients with myofascial pain syndrome.

## Introduction

According to the United States Centers for Disease Control and Prevention, the three main conditions that cause daily life disability are heart disease, arthritis, and chronic back pain (1). Myofascial pain syndrome (MPS) is a non-inflammatory syndrome that presents with symptoms of muscle pain and limited range of joint motion. MPS occurs in 11.9–44.8% of patients who complain of back pain (2, 3). There is currently no standard treatment for patients with MPS (4). Clinically available treatments are trigger point injections of a local anesthetic, dry needling, manual therapy, physical exercise, and self-myofascial release (5–8).

Ultrasound-guided hydrodissection has recently been receiving increased attention as a therapy for treating MPS (9, 10). In ultrasound-guided hydrodissection, doctors use ultrasound to inject a drug solution into the interfascial space (including subcutaneous tissue, epimysial space, the space between the periosteum and fascia, and the periphery of tendon) at the site where patients feel the most severe pain. The site is located at the periphery of the muscle diagnosed with MPS. The mechanism underlying the clinical efficacy of the method is unclear; however, pressure stimulation with drug solution injection, washout of pain-inducing substances, and acid-induced stimulation of acid sensing channels have been suggested. The saline is often administered instead of local anesthetics in Japan because the analgesic effect by anesthesia in not expected.

We previously reported that MPS is a clinical symptom of psychosomatic disorder that occurs in approximately half of cancer patients. The results from a randomized controlled study suggested that biofeedback therapy, a psychosocial approach, is helpful in the treatment of MPS in cancer patients (11). Clinically, visual feedback during ultrasound-guided hydrodissection is expected to be effective as psychosocial approaches, whereby patients raise their expectations for treatment through real-time viewing of their ultrasound image (during the removal of fascial adhesions by saline injection) with doctors while receiving hydrodissection. However, to date, there have not been any studies that have investigated whether visual feedback during ultrasound-guided nerve blocks, such as hydrodissection, is effective as a psychosocial approach.

Expectations for treatment have a favorable effect on the subsequent course and behavior in patients. A systematic review has suggested that positive expectations for treatment are related to good health outcomes (12). Furthermore, expectations for treatment have shown to play an important role in the placebo effect (13). A previous study reported that positive expectations for pain treatment has a positive effect on the subsequent course of pain and behavior in patients (14).

We hypothesized that visual feedback immediately after hydrodissection would raise patients’ expectations for pain treatment, which could be one of the related factors for pain reduction. To the best of our knowledge, there have not been any reports that have investigated the effect of visual feedback during ultrasound-guided hydrodissection for MPS.

## Materials and methods

### Objective

The objective of this study was to explore the possibility that visual feedback immediately after hydrodissection increases the expectations for treatment, which could be one of the related factors for pain reduction.

### Study design

This was an exploratory, prospective, observational clinical trial that explored the effects of visual feedback during ultrasound-guided hydrodissection. Visual feedback was defined as hydrodissection administration while patients viewed the ultrasound image with their doctor. Expectations for treatment were set as mediators of pain using path analysis.

### Ethics statement

The study received approval from the Medical Ethics Committee of Kansai Medical University on March 6, 2019 (reference number: 2018177). Informed consent was not obtained in this study because usual clinical practice was observed, including assessments and treatment. An opt-out method was used so that patients and their families could refuse to participate in the study. The procedures performed in this study were in accordance with the Declaration of Helsinki (as revised in 2013). This study was registered with the University hospital Medical Information Network Clinical Trials Registry (approval number: UMIN000043160) on January 28, 2021 (retrospectively registered).

### Study participants and eligibility criteria

This study was conducted from March 2019 to March 2020 at two facilities in Japan: Kansai Medical University Hospital and Omotesando Pain Clinic. During this period, we continuously enrolled outpatients who received ultrasound-guided hydrodissection for MPS of the upper back. MPS was diagnosed based on the following criteria: (1) a tender spot located with palpation, with or without referral of pain; (2) recognition of symptoms by the patient during palpation of the tender spot; and (3) at least three of the following: (a) muscle stiffness or spasm, (b) limited range of motion (ROM) of an associated joint, (c) pain worsening with stress, and (d) palpation of a taut band and/or nodule associated with the tender spot (15). The upper back was defined as the region below the neck and above the costal margin (16).

The exclusion criteria were: (1) patients who were younger than 20 years and (2) patients who had any comorbid psychiatric disease or condition that made communication difficult, such as cognitive impairment or delirium.

### Procedure

**FIGURE 1:**
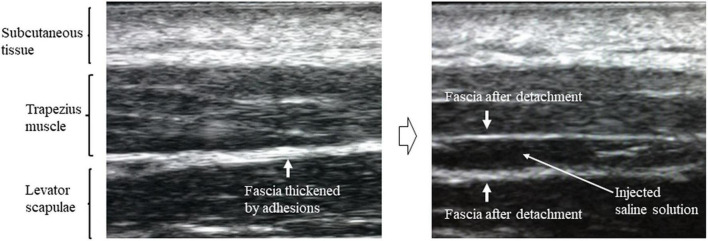
Ultrasound image showing thickening of the fascia between the levator scapulae and trapezius muscles before hydrodissection and removal of fascia after hydrodissection.

Hydrodissections (5 ml saline per injection) were administered by a doctor at each institution to four bilateral upper and lower sites at the boundary between the levator scapulae and trapezius muscle. Doctors used ultrasound to confirm the following points in real-time: (1) boundary between the levator scapulae and trapezius muscle; (2) position of the needle tip; (3) saline injection; and (4) removal of the fascia (Figure 1). A single hydrodissection session was performed on the day of intervention.

Before starting treatment, a doctor at each institution verbally explained the ultrasound-guided hydrodissection procedure [i.e., points (1) to (4) above] to the patients. Patients were then asked whether they would like to request to view the real-time ultrasound images during the procedure. The merits and demerits of visual feedback were not shared with the patients. Patients who viewed the real-time ultrasound images were defined as the visual feedback group, and patients who did not view the real-time ultrasound images were defined as the non-visual feedback group. The visual feedback group received the doctor’s explanation of the procedure and ultrasound images with viewing the real-time ultrasound images; the non-visual feedback group received the doctor’s explanation of the procedure and ultrasound images without viewing the ultrasound images. Both groups spent equal amounts of time in each session. After the ultrasound-guided hydrodissection, patients were instructed to perform two 3-min sessions of a self-stretching exercise (right-left cervical rotation) per day. Assessments were carried out by a doctor in the outpatient department or clinic of each institution on the following days: before intervention (T0), the day of the hydrodissection (T1), and 14 days after the hydrodissection (T2).

### Measures

#### Clinical demographic characteristics

Clinical demographic information was obtained from all subjects and included age, sex, institution (hospital or clinic), primary illness (cancer or non-cancer), pain numerical rating scale (NRS) before the hydrodissection, NRS to assess expectations for treatment before hydrodissection, and analgesic drug use.

#### Measures of expectations for treatment and criteria for increased expectations

Expectation intensity was determined using an NRS to assess expectations for treatment, which ranged from 0 (no expectations) to 10 (highest expectations). The questionnaire was self-administered and contained the following question: “How well do you expect this treatment to reduce pain?” The validity of this questionnaire is not clear, but it has frequently been used in research (12, 14). The criterion for increased expectations was determined as an NRS score ≥8 or ≥33% improvement in NRS score for expectations for treatment after hydrodissection.

#### Measures of pain intensity and criterion for pain reduction

Average pain intensity was assessed using an 11-point NRS for pain, which ranged from 0 (no pain) to 10 (worst possible pain) (17). The questionnaire was self-administered and contained the following question: “How intense was your average pain over the past 24 hours?” For patients with multiple MPS sites, we used the average pain NRS score. For pain at T1, pain intensity was evaluated as pain at the time. The reliability and validity of this scale have been established previously (18). The criterion for pain reduction was determined as ≥50% improvement in pain NRS score after the intervention. The optimal cut-off point for NRS rate of change has been reported to be 50% when determining the proportion of patients with clinically significant pain improvement (19).

#### Measures of cervical range of motions

Cervical ROM for flexion, extension, lateral flexion, and rotation were measured using a goniometer (TAKUMED, Kyoto, Japan), which is an objective and reliable method (20). All measurements were obtained by one doctor at each institution.

### Outcomes

The primary endpoint was the NRS to assess expectations for treatment immediately after ultrasound-guided hydrodissection, between with and without the visual feedback. The secondary outcomes were the proportion of increased expectations immediately after hydrodissection, NRS score change for expectations for treatment and pain, the proportion of pain reduction at T2 after hydrodissection, cervical ROM, correlation between increased expectations and pain NRS reduction using path analysis, and adverse events.

### Sample size calculation

Because previous studies on this subject are limited, sample size calculation was performed based on a report using similar therapeutic methodologies (21). The primary endpoint was the NRS to assess expectations for treatment immediately after visual feedback during ultrasound-guided hydrodissection, while one of the outcome of the previous report was the NRS to assess expectations for treatment immediately after the immediate effect of trigger point injection with local anesthetic. The previous study showed that a NRS to assess expectations for treatment before the immediate effect of trigger point injection was 5.1 (standard deviations, 2.3), whereas it increased to 7.5 (standard deviations, 2.3) immediately after the immediate effect. A NRS to assess expectations for treatment before the non-immediate effect was 4.9 (standard deviations, 2.4), and after the non-immediate effect was 5.7 (standard deviations, 2.9). We assumed an NRS to assess expectations immediately after hydrodissection with and without visual feedback are 7.5 (standard deviations, 2.5) and 5.8(standard deviations, 2.5), respectively. The sample size required to achieve 95% statistical power at a 5% two-sided significance level was 62 patients per group. Considering 10% rate of withdrawal, we determined a total sample size of 136 patients.

### Statistical analysis

Data are reported as means and standard deviations, medians with interquartile ranges, or frequencies (%), as appropriate. When participants provided missing data, we used the worst scores in the data.

The study participants were classified into two groups: the visual feedback and non-visual feedback groups. Unpaired *t*-tests were used for comparisons of the independent variables of age, pain NRS score before hydrodissection, and NRS score for expectations for treatment before hydrodissection. Pearson’s chi-square tests were used to analyze the independent variables of sex, institution, primary illness (cancer), and analgesic drug use. The proportion of study participants with visual feedback, increased expectations immediately after hydrodissection, and pain reduction at T2 after hydrodissection for each group among all participants were estimated using a chi-square test, including the exact 95% confidence intervals (95% CI).

Changes in the course (T0, T1, and T2) of NRS scores for expectations for treatment, pain NRS scores, and cervical ROM scores were analyzed using one-way repeated measures analyses of variance (ANOVA) for each group. To conduct comparisons between groups, time course was used as the within-subjects factor and group was used as the between-subjects factor in a two-way repeated measures ANOVA. Multiple comparisons were corrected using the Bonferroni method. If participants withdrew from the study, NRS scores after withdrawal were substituted with scores immediately before withdrawal. Change in analgesic drug use during the period and loss to follow up were classified as withdrawals from the study.

**FIGURE 2:**
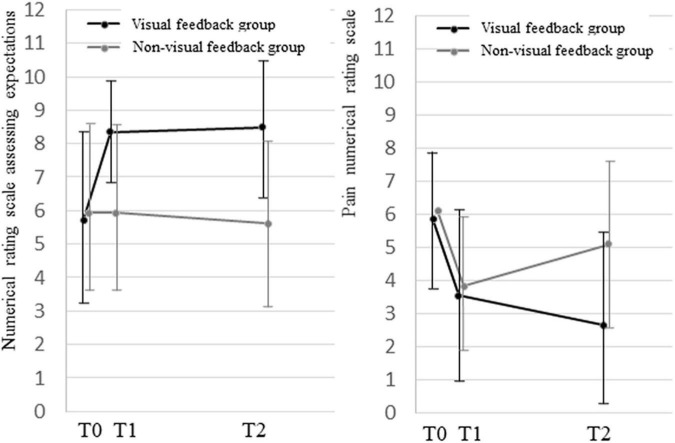
Path diagram for the hypothetical model. NRS, numerical rating scale.

The traditional path analyses were conducted to estimate the direct and indirect paths with reference to correlation coefficients. A hypothetical model was created in which visual feedback, pain reduction at T1, expectations at T0, and increased expectation at T1 predicted pain reduction at T2. Visual feedback, age, analgesic drug use, NRS at T0, expectations at T0, pain reduction at T1, and increased expectations at T1 were mediators of increased expectations and pain reduction at T2. Figure 2 shows the hypothetical model [Akaike information criterion (AIC) = 116.832]. Path analyses were performed by removing paths with *p* < 0.05, adjusting paths with reference to the modification index, repeating model correction while checking the goodness of fit index (GFI), and investigating correlations between factors specifying pain reduction at T2. To assess fit, we used model chi-square values, GFI, comparative fit index (CFI), root mean square error of approximation (RMSEA), and AIC. Smaller chi-square values, >0.95 for GIF and CFI values, and ≤0.08 RMSEA values indicate good model fit (22). The AIC was used to compare the hypothetical model with the modified model; a lower AIC value indicated a better model.

A value of *p* < 0.05 was considered statistically significant. Statistical analyses were performed using SPSS version 25.0 and Amos version 25.0 for Macintosh (SPSS, Inc., IBM, Chicago, IL, United States).

## Results

### Number of registered study participants

During the study period, 149 outpatients who received ultrasound-guided hydrodissection for MPS were enrolled. Of these 149 outpatients, 13 patients were excluded for the following reasons: (1) aged <20 years (*n* = 5) and (2) had a comorbid psychiatric disease or condition that made communication difficult (*n* = 8). A total of 136 patients were selected as study participants.

### Clinical demographic characteristics

Of the 136 patients, 47.8% (95% CI: 39.4–56.2) received visual feedback during ultrasound-guided hydrodissection. Patients were classified into the visual feedback (*n* = 65) or non-visual feedback (*n* = 71) groups. Table 1 shows the clinical demographic and characteristics of each group.

**TABLE 1:** Clinical characteristics of the visual feedback and the non-visual feedback groups.

	Visual feedback group (*n* = 65)		Non-visu al feedback group (*n* = 71)		*P*-value
	Mean	SD	Mean	SD	
Age, years	60.5	(15.3)	59.9	(15.9)	0.847
	***n***	**%**	***n***	**%**	
			
Sex, female	33	(50.8)	42	(59.2)	0.209
Institution, hospital	45	(69.2)	50	(70.4)	0.514
Primary illness, cancer	44	(67.7)	51	(71.8)	0.367

	**Mean**	**SD**	**Mean**	**SD**	
Pain NRS score (before IFI)	5.9	(2.1)	6.1	(2.2)	0.493
NRS score assessing expectations for treatment (before IFI)	5.7	(2.6)	5.9	(2.5)	0.615

	**n**	**%**	**n**	**%**	
			
Analgesic drug use					
None	25	(38.5)	28	(39.4)	0.524
Use	40	(61.5)	43	(60.6)	
Non-opioid	21	(52.5)	18	(41.9)	0.288
Opioid use	19	(47.5)	25	(58.1)	
	**Median**	**IQR**	**Median**	**IQR**	
			
Opioid dose (mg/day)*^a^*	30	(30, 60)	30	(20, 60)	

NRS, numerical rating scale; SD, standard deviation; IQR, interquartile range.

^a^Dose of opioids is expressed as oral dose level of morphine(mg/dl). For conversion: parenteral morphine: oral morphine = 1:2, parenteral, oxycodone: oral morphine = 1:2, oral oxycodone: oral morphine = 2:3, fentanyl: morphine = 1:100, oral methadone: oral morphine = 1:8.

Ten patients withdrew from the study because of analgesic drug changes during the study period (*n* = 3) or loss to follow up (*n* = 7). Of these patients, four were in the visual feedback group and six were in the non-visual feedback group.

### Numerical rating scale to assess expectations for treatment and the proportion of increased expectations immediately after hydrodissection

In the visual feedback group, a NRS to assess expectations for treatment at T0 was 5.7 (standard deviations, 2.6), whereas it increased to 8.4 (standard deviations, 1.6) at T1. In the visual feedback group, a NRS to assess expectations for treatment at T0 was 5.9 (standard deviations, 2.5), and at T1 was 5.9 (standard deviations, 2.6). The proportion of increased expectations immediately after hydrodissection was 90.8% (95% CI: 83.7–97.9) in visual feedback group and 38.0% (95% CI: 26.7–49.3) in non-visual feedback group (*p* < 0.001).

### Between-group comparison of numerical rating scale score change for expectations for treatment and pain

**FIGURE 3:**
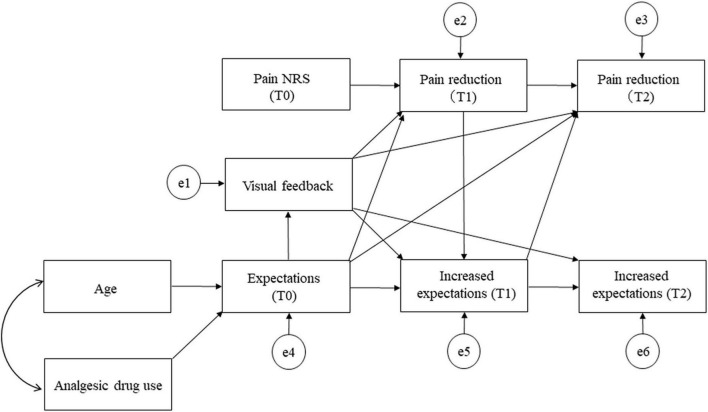
Between-group comparison of numerical rating scale score change for expectations for treatment and pain.

The comparison of NRS score change for expectations for treatment between groups showed a significant interaction between time course and group (*p* < 0.001; Figure 3). Compared with the non-visual feedback group, the visual feedback group had higher expectations for treatment immediately after hydrodissection, and the higher expectations were maintained at T2. There was a significant difference in time course between the two groups at T0 and T1, T2 (*p* < 0.001; *p* < 0.001; Figure 3).

The comparison of NRS score change between groups showed a significant interaction between time course and group (*p* < 0.001). There was no significant difference for T1 between the two groups (*p* = 0.186). The visual feedback group showed a significant decrease in NRS score at T2 (*p* < 0.001; Figure 3).

### The proportion of pain reduction at T2 after hydrodissection

In the visual feedback group 67.7% of patients showed improvement in pain numerical rating scale score by 50% or more at day 14 (95% CI: 56.5–78.9), whereas such improvement was observed in only 36.6% in the non-visual feedback group (95% CI: 25.3–47.9; *p* < 0.001). Overall, improvement was observed in 52.2% of patients (95% CI: 43.8–60.6).

### Within-and between-group comparisons of cervical range of motion score change

In both groups, all measures of cervical ROM improved over time. There was a significant difference in time course between the two groups for right (*p* = 0.028) and left rotations (*p* = 0.019; Table 2).

**TABLE 2:** Cervical range of motion scores in the visual feedback and non-visual feedback groups and the comparison of differences within and between group.

	Visual feedback group					Non-visual feedback group					
	*n* = 65					*n* = 71					
											*P*-value
	T0		T2			T0		T2			
					*P*-value					*P*-value	
	Mean	SD	Mean	SD		Mean	SD	Mean	SD		
Flexion	41.5	8.8	46.5	8.9	<0.001	39.1	11.5	42	9.6	0.036	0.393
Extension	41.2	9.8	46.8	7.3	<0.001	43.1	10.9	46.3	10.9	0.001	0.291
Lateral flexion (right)	30.5	8.6	35.5	10.9	<0.001	31.1	9.1	33.9	9.6	0.001	0.333
Lateral flexion (left)	31.9	9.2	35.2	11.8	0.002	31.3	9.4	33.5	10.6	0.012	0.675
Rotation (right)	47.1	15.0	60.2	12.1	<0.001	46.6	18.6	51.2	16.8	<0.001	0.028
Rotation (left)	45.4	14.1	58.5	10.4	<0.001	47.0	15.3	52.3	14.6	<0.001	0.019

SD, Standard deviation.

### Path diagram for the final model

**FIGURE 4:**
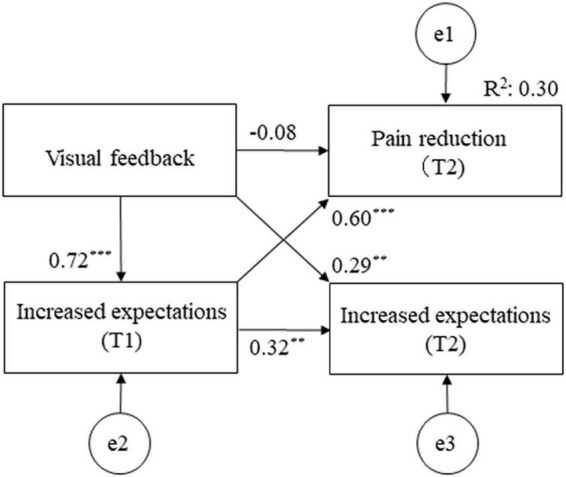
Path diagram for the final model.

The final model fit the data well (model chi-squared value = 0.001, CFI = 1.000, RMSEA = 0.000, and AIC = 26.001; Figure 4). Visual feedback had the most influence on NRS reduction at T2 (β = 0.356). Furthermore, visual feedback had the greatest influence on pain reduction at T2, indirectly due to increased expectations for treatment after ultrasonic-guided hydrodissection as a mediator (β = 0.434). The adjusted *R*^2^ values, which is the overall fit of the model, was 0.298.

### Adverse events

There were no adverse events due to ultrasound-guided hydrodissection. In addition, none of the patients in the visual feedback group experienced adverse events, such as feeling unwell.

## Discussion

The first critical point of this study was the positive effect of visual feedback during ultrasound-guided hydrodissection on expectations for treatment. Fifteen percent of psychosocial approach effects have been attributed to placebo effects which depend on patients’ expectations for treatment (23). To the best of our knowledge, this study is the first to investigate the effect of visual feedback during ultrasound-guided hydrodissection as a psychosocial approach using an exploratory, prospective observational study design. Placebo effects should be used actively as much as possible in clinical practice to improve patients’ expectations for treatment and therapeutic outcomes (24).

In the visual feedback group, patients’ expectations for treatment increased significantly immediately after the intervention. In a study on family caregivers of cancer patients, patients who became aware of comfort immediately after the introduction of relaxation had significantly higher expectations for treatment immediately after intervention compared with those of the control group (25). Another study in advanced cancer patients with dizziness showed that awareness of muscle relaxation during hypnosis increased both expectation for treatment and implementation rate of self-care (26). However, our study suggested that patients’ expectations for treatment increased through awareness of vision during ultrasound-guided hydrodissection.

In the visual feedback group, cervical ROM for rotation significantly improved in patients who were instructed to perform self-stretching exercises. However, other measures of cervical ROMs (flexion, extension, and lateral flexion) did not differ between patients who were and were not instructed to perform self-stretching exercises. These results indicated that in the visual feedback group, an increase in patients’ expectation for treatment immediately after the intervention led patients to adhere more to self-stretching.

The second critical point was that the expectations for treatment could be one of the determining factors for pain reduction. The severity of upper back pain in MPS was 6.0 on the NRS, which reflects moderate pain (27). At 14 days after ultrasound-guided hydrodissection, there was a relatively high proportion of patients who achieved ≥50% improvement in pain NRS scores. However, because there have not been any similar studies conducted previously, we cannot compare this result with other findings. One study reported that ≥50% improvement in pain NRS score was observed in 48.8% of cancer patients with MPS, 1 week after receiving trigger point injections of a local anesthetic (2). In our study we observed a 67.7% improvement rate in the visual feedback group, which is higher than the abovementioned study. Thus, visual feedback, a simple yet ingenious device, can offer considerable relief of moderate pain in patients with MPS, which is of clinical significance. When considering the potential for an increase in subsequent analgesic effect on patients with MPS who request visual feedback treatment for upper back pain, our results may suggest evidence to recommend visual feedback treatment. Because our study was an observational study, negative effects could not be assessed for patients with MPS who received visual feedback treatment despite declining treatment.

Visual feedback had no direct effect on the subsequent course of pain. However, it had an indirect positive effect through increased expectations for treatment. We speculate that a placebo effect on pain in MPS patients was exerted by patients’ expectations for treatment. A previous study reported that opioid-naïve cancer patients with high expectations for pain reduction before morphine treatment had significantly lower pain intensities at 7 days after treatment (14). There have been two further reports that have indicated that the placebo effect is easily obtainable, particularly for pain in patients with MPS. One study investigated cancer patients with MPS (2), which showed that the proportion of cancer patients with MPS who also experienced psychological stress was 57.2%; the response rate to trigger point injection in these patients was significantly higher than that of cancer patients with MPS without psychological stress. The second study was based on MPS in family caregivers of cancer patients (28), which found that relative factors vary by back region and that MPS in the upper back is more easily affected by psychological stress. Therefore, the authors recommended psychosocial approaches for treating MPS in the upper back.

We also speculate that in the visual feedback group, the immediate increase in patients’ expectations for treatment after the intervention increased the adherence rate of subsequent self-stretching, which resulted in MPS pain relief 14 days after the intervention. Efficacy of self-stretching on MPS in the plantar heel and stretching plus acupuncture on MPS in the upper back have been reported previously (29, 30).

However, path analysis revealed a low adjusted *R*^2^ value at 0.298, which measures the overall fit of the model. In other words, visual feedback and increased expectations explain only 30% of the pain reduction“Increased expectations” is the only one among several other factors associated with pain reduction.

### Study limitations

The study has several limitations. First, self-selection bias was present because visual feedback was not allocated. Patients with high expectations for treatment may have requested visual feedback treatment. However, there was no significant difference between the two groups in NRS scores for expectations for treatment before hydrodissection. This study was an exploratory trial, and future randomized controlled trials with or without visual feedback with expectations for treatment as the primary endpoint are needed. Second, hydrodissection is a new treatment and has not been established as a standard treatment for MPS. Therefore, these results are limited to the method of this study. Third, our results may not generalize to the general population because there was a large proportion of cancer patients. Although a recent review reported a high frequency of MPS in cancer patients (3), cancer patients are uncommon population as a clinical research of MPS. Forth, we did not investigate whether there was a difference between group in the awareness of recurrent pain due to the spread of the injectate on interfascial space, which may provide patients’ expectations. The awareness of pain changes immediately after trigger point injections has been reported to influence expectations (21). Fifth, the timing of maximum effect and disappearance was obscure because of the two-point test (the day and 14 days after intervention). It seemed to be effective in the short term, but no long-term effect was found. Even at 14 days after intervention, a more potent analgesic effect was probably attainable because patients’ expectation for treatment remained high. Finally, the effect repeated visual feedback effect was not known because of the single session. We expect that the effects will be influenced by increased expectations before the next session.

## Conclusion

Visual feedback during ultrasound-guided hydrodissection increases the expectations for treatment immediately after hydrodissection, which could be one of the related factors for pain reduction in patients with myofascial pain syndrome.

## Data availability statement

The raw data supporting the conclusions of this article will be made available by the authors, without undue reservation.

## Ethics statement

The studies involving human participants were reviewed and approved by the Ethics Committee of Kansai Medical University. Written informed consent for participation was not required for this study in accordance with the national legislation and the institutional requirements.

## Author contributions

HH was responsible for the conception and design of this study, wrote the manuscript, and responsible for data analysis. HH and HO were responsible for data collection and for clinical evaluations. MF provided advice on the composition of the manuscript. All authors have approved the final version of this manuscript.

## Abbreviations

MPS: myofascial pain syndrome
ROM: range of motion
NRS: numerical rating scale
95% CI: 95% confidence intervals
ANOVA: analysis of variance
AIC: Akaike information criterion
GFI: goodness of fit index
CFI: comparative fit index
RMSEA: root mean square error of approximation.
